# Methodological considerations for the force-matching task

**DOI:** 10.3758/s13428-022-01954-w

**Published:** 2022-08-24

**Authors:** David McNaughton, Rhys Hope, Emily Gray, Freya Xavier, Alissa Beath, Michael Jones

**Affiliations:** School of Psychological Sciences, Macquarie University, Balaclava Road, Sydney, Australia

**Keywords:** Sensory attenuation, Perception, Sensorimotor, Force-matching, Methodology

## Abstract

**Abstract:**

The force-matching task integrates haptic technology and electrical engineering to determine an individual’s level of sensory attenuation to somatic stimuli. The task requires a detailed methodology to facilitate reliable and replicable estimates, and there has been a distinct lack of re-evaluation of the methodological processes related to this paradigm. In this task, participants are asked to match a force delivered to their finger, either by pressing directly on their own finger with their other hand (known as the direct condition) or by controlling the device using an external potentiometer to control the force indirectly through a torque motor (known as the slider condition). We analysed 138 participants to determine 1) the optimal number of replications (2, 4, 6, or 8 replications) of the target force, 2) the optimal time window (1–1.5 s, 1.5–2 s, 2–2.5 s and 2.5–3 s) to extract the estimate of sensory attenuation, 3) if participants’ performance during the task improved, worsened or was stable across the experimental period regardless of condition, and 4) if learning effects were related to psychological traits. Results showed that the number of replications of the target forces may be reduced from 8 without compromising the estimate of sensory attenuation, the optimal time window for the extraction of the matched force is 2.5–3 s, the performance is stable over the duration of the experiment and not impacted by the measured psychological traits. In conclusion, we present a number of methodological considerations which improve the efficiency and reliability of the force-matching task.

**Highlights:**

• The force-matching task determines an individual’s level of sensory attenuation

• The optimal number of replications of the target force may be reduced from 8

• The optimal time window to extract the matched force is 2.5–3.0 s

• The estimate of sensory attenuation is stable across the duration of the task

## Introduction

It is important for the human somatosensory system to effectively distinguish, and attend to, sensory information that carries greater evolutionary importance (Brown et al., 2013). One neural process by which this occurs is during the prediction of sensory consequences of self-generated action (Wolpert & Flanagan, 2001). An example of this is during human movement, where internal models predict the sensory outcome of actions via a copy of the motor command. An important component of this process is that the predicted sensory information associated with movement is compared with the actual sensory feedback, and this partially cancels out sensory consequences of self-generated movement (Bays et al., 2005). This process is termed perceptual sensory attenuation and is defined as a reduction in the perception of the afferent input of a self-produced tactile sensation due to the central cancellation of the reafferent signal by the efference copy of the motor command to produce the action (Palmer et al., 2016).

Sensory attenuation can be measured via an experimental paradigm known as the force matching task (Shergill et al., 2005). In this task, participants are asked to match a force delivered to their finger, either by pressing on their own finger, via the device lever with their opposite hand (known as the direct condition) or by controlling the device using an external potentiometer to control the force indirectly through a torque motor (known as the slider condition). In both conditions, the force produced is known as the matched force. In previous research, it has been shown that healthy people consistently generate a greater matched force and tend to overestimate the force in the direct condition when compared to the slider condition (Wolpe et al., 2016). The excess force produced in the direct condition reflects the sensory attenuation phenomenon. Deficits in sensory attenuation measured via the force-matching has been identified in individuals with schizophrenia (Shergill et al., 2005) and functional motor syndromes (Parees et al., 2014). This has been theoretically related to altered predictive mechanisms leading to a loss of agency (Brown et al., 2013).

The force-matching task requires a device which integrates haptic technology and complex electrical engineering. A number of research teams have designed their own custom made devices (Shergill et al., 2005; Valles & Reed, 2013; Walsh et al., 2011) or recommissioned existing haptic technology (Parees et al., 2014). A challenge for this research paradigm, then, has been consistency across different groups in terms of force delivery and measurement of force applied by participants. Previous work by our group has published the blueprints and software necessary to build such a device, in an effort to improve the diversity of research in this area (McNaughton et al., 2021). Although the principles of the paradigm are straightforward, the force-matching paradigm requires a detailed and transparent methodology to facilitate a reliable and replicable estimate of an individual’s level of sensory attenuation.

There has been a distinct lack of re-evaluation of the methodological processes related to this paradigm. The key outcome variable of paradigm is the mean force error, which is calculated as the matched force minus the target force. This estimate is aggregated across several replications of the target forces and extracted from a 500-ms time interval while the participant attempts to reproduce the target force. Due to the cognitively demanding nature of the paradigm, learning effects over the course of the experimental are possible but have not been studied. Therefore, it is the purpose of the current study was to investigate these methodological research questions to improve the efficiency and reliability of the force-matching task.

It has been proposed that it is necessary to replicate each target force a minimum of eight times in each condition in order to gain a reliable estimate (Parees et al., 2014; Wolpe et al., 2016). This results in 64 trials (4 forces x 8 replications x 2 conditions). However, while eight replications has been argued for, the optimal number of replications of the matched force has not been empirically studied to our knowledge. If the number of replications of the target force could be reduced without compromising our estimates of sensory attenuation, this would lead to a more efficient paradigm and consequently less fatigue in participants.

Another key methodological question arises from the specific time window for the extraction of the matched force. This time window has differed between research groups, with some using 2.0–2.5 s after presentation of the target force (Shergill et al., 2005; Teufel et al., 2010; Voss et al., 2007), and others between 2.5–3.0 s (Palmer et al., 2016; Parees et al., 2014). It is critical to determine the time window that experiences the least amount of fluctuation in the matched force.

Finally, considering the length of time necessary to conduct to the minimum replications and time window for the extraction of the matched forces, it is unknown whether any learning or fatigue effects occur. This would be evident through signs of improvement, stability in performance, or declining performance over the course of the experiment. Further, there is evidence to suggest an individual’s level of sensory attenuation may be correlated with psychological constructs, such as delusional ideology (Teufel et al., 2010). Therefore, a related aim is to determine any moderating impacts on participant learning or fatigue by psychological traits.

In summary, the aims of the current paper are to 1) determine the stability of the mean force error across different replications of the target force, 2) determine the optimal time window for the extraction of the mean force error, 3) identify whether learning or fatigue effects associated with matching a force over the course of the experiment, and 4) whether learning or fatigue effects are moderated by any psychological traits of the participant.

## Methods

### Participants and setting

A total of138 right-handed participants took part in a single experimental session which incorporated the force-matching task and completion of a questionnaire determining demographic and self-reported psychological functioning. The study was approved by the Macquarie University Human Sciences Research Ethics Subcommittee (approval number: 52019574612789).

### Force-matching task

A detailed description of the force-matching device design, functionality and task can be found in McNaughton et al. (2021).

The force-matching task consisted of two conditions: a) The direct condition, in which participants match a target force by pressing directly on top of a lever, mechanically transmitting the force to the right index finger; and b) the slider condition, in which participants match the force using their left index finger by moving a slider (potentiometer), which controls the torque motor. Each participant was asked to reproduce four separate forces (1, 1.5, 2, and 2.5 N) on eight separate trials in a randomised order under both direct and slider conditions. The order of condition (total block of 32 trials) was counterbalanced across participants and short 1-min pause was provided to participants after every 16 trials. In the direct condition, the device exerted one of the four constant target forces in each trial for 3 s. After 2 s of rest, an auditory “go” signal instructed the participants to start matching the target force by directly pressing with their left index finger for 4 s onto the force transducer resting on the right index finger. A “stop” auditory signal marked the end of the trial. In the slider condition, the device exerted one of the four constant target forces in each trial for 3 s. After 2 s of rest, an auditory “go” signal indicated the participant to start matching the target force by moving the external potentiometer with their left finger. This controlled the output of the torque motor that applied a force to the right index finger. A force sensor at the end of the lever measured both the target and matched forces applied to the right finger.

### Self-reported psychological health measures

Four dimensions of psychological health of participants were evaluated: depression, anxiety, state affect and delusional ideology. These measures were used to determine whether learning or fatigue effects were moderated by psychological traits of the participants. Depressive symptomology was measured with the nine-item Patient Health Questionnaire (PHQ-9) (Kroenke et al., 2001). Each item on the PHQ-9 is scored from 0 to 3, with a total score ranging from 0 (no depressive symptomology) to 27 (high levels of depressive symptomology). Anxiety was measured with the seven-item Generalized Anxiety Disorder Questionnaire (GAD-7). Each item on the GAD-7 is scored from 0 to 3, with a total score ranging from 0 (no anxiety) to 21 (high levels of anxiety). Acceptable psychometric properties of the PHQ-9 (Kroenke et al., 2001) and GAD-7 (Kroenke et al., 2016) are well established. Negative and positive affect were measured with the Positive and Negative Affect Schedule (PANAS), a reliable and well validated instrument (Crawford & Henry, 2004) consisting of ten positive and ten negative statements (Watson et al., 1988). Participants were asked to indicate the extent their feelings corresponded to the words in the past week on a five-point scale, with a total score ranging from 10 (low negative or positive affect) to 50 (high negative or positive affect). Delusional ideology was measured using the Delusion Inventory (Peters et al., 2004). This consisted of 21 statements in which participants had to respond using a “yes/no” binary scale. This was designed to quantify delusion-like ideas in the general population. A total score was calculated (0–21) with high scores reflecting high levels of delusional ideology.

### Statistical analyses

The mean force error was calculated as mean matched force minus the target force. We separately calculated composites scores by aggregating the error values across target forces. This gives a single error value in each condition. We investigated if there was any difference between the direct and slider conditions by using Wilcoxon rank-sum tests. These analyses provided context to the overall performance of the task by participants and the same data have been reported on a smaller sample size in McNaughton et al. (2021). Reliability estimates of the mean force error were calculated via the Spearman–Brown formula in both the direct and slider conditions. All analyses were performed using STATA v16 (StataCorp, 2017). The associated data and analyses code can be obtained here: 10.25949/19694998.v1.

Statistical significance testing reported in this manuscript is a combination of tests of a priori hypotheses concerning variation in outcome with experimental methodology-related factors and exploratory analyses concerning variation in outcome with individual difference factors. Where necessary statistical significance was determined using the Bonferroni approach for a priori hypothesis testing to maintain an overall type I error rate of 0.05, although as it transpired this was not necessary. A *p* value < 0.05 was considered statistically significant for the exploratory analyses and we acknowledge that these findings need replication in future, independent samples.

#### Aim 1 – Determine the stability of the mean force error across different replications of the target force

Each participant matched a target force eight times, for four different forces (1, 1.5, 2 and 2.5 N) and in two conditions (direct or slider). Thus, a total of 64 trials (8×4×2 = 64) per participant were conducted. The mean force error estimates were calculated from the first 2, 4, 6, and 8 trial replications, respectively, for each force and condition. Within-subject analysis of variance (ANOVA) were conducted to contrast the mean force error (as the dependant variable) and number of replications (as the independent variable). A total of eight analyses were conducted which included an omnibus test of simultaneous equality of all number of replications of the target force, as well as specific contrasts of eight replications with each of the 2, 4, and 6 replications. These contrasts were conducted for each condition and target force separately. Finally, reliability estimates of the mean force error were calculated via the Spearman–Brown formula in both the direct and slider conditions for 2, 4, and 6 replications of the target force. This was done to allow comparison of reliability estimates with the standard eight replications.

#### Aim 2 – Determine the optimal time window for the extraction of the mean force error estimates

To determine the optimal time window to extract the match force, each participant had the average force applied to the sensor recorded over four different intervals following the go signal: 1–1.5s, 1.5–2s, 2–2.5s and 2.5–3s. The initial 1s of matched force was not included in the analysis as previous literature (and our own experience) has identified this time-period to be highly variant within and between participants (Voss et al., 2007), thus not relevant when determining the most optimal and stable time window for extraction. Mean force error estimates were calculated for each time window, for each target force and condition. Within-subject repeated measure ANOVA were conducted to contrast the mean force error (as the dependent variable) against time window (as the independent variable). A total of 8 analyses were conducted which included an omnibus test of simultaneous equality of all time windows, as well as specific contrasts of the 2.5–3.0-s time window with each 1–1.5-s, 1.5–2-s and 2–2.5-s time windows. These contrasts were conducted for each condition and target force.

#### Aim 3 – Determine learning effects associated with matching a force over the course of the experiment

To determine learning effects or fatigue throughout the task, we calculated the mean force error in each of the 64 trials. A coefficient representing the slope of the mean force error of these trials was then calculated via a linear regression analysis for each subject. This is termed the mean force error coefficient (also represented as the slope). We presented the mean force error coefficient and the distribution of this coefficient showing the extent of individual variation. We considered the magnitude and statistical significance of the individuals’ coefficients and deemed the statistical significance as not relevant to the study aim, and thus only reported the magnitude.

#### Aim 4 – Determine whether learning or fatigue effects are moderated by any psychological traits of the participant

To determine whether learning/fatigue effects were moderated by psychological traits, spearman correlations evaluated the relationship between psychological constructs of anxiety, depression, affect (positive and negative), and delusion ideation with the mean force error coefficient.

## Results

**Fig. 1:**
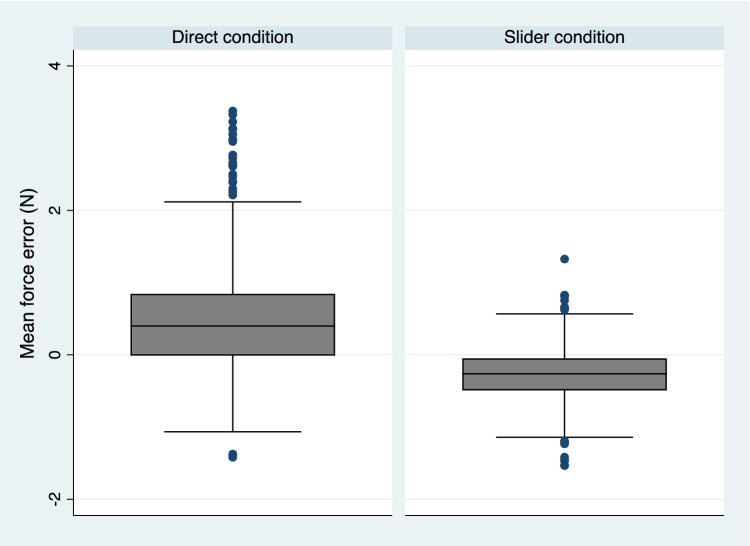
Standard box plots showing the distribution of mean force error values across participants in the direct and slider conditions. A statistically significant difference was determined between the direct and slider conditions

Table 1 displays the sample demographics. The sample was slightly more female and were considered normal on all self-reported psychological measures. Of the 138 participants, the mean force error in the direct condition was 0.47 N (SD = 0.76) and was statistically significantly greater than zero (z = 7.22, *p* < 0.001). Within the slider condition the mean force error was smaller – 0.27 N (SD = 0.38) and this was statistically significantly less than zero z = – 8.08, *p* < 0.001. The overall mean difference between the direct and slider with respect to the error was 0.76 N (SD = 0.67) and this was statistically significantly different (z = 10.40, *p* < 0.001), indicating that a greater force was applied in the direct condition denoting a clear difference in sensory attenuation and perception of the target forces. Figure 1 displays box plots of the mean force error by condition, highlighting this difference. Split-half reliability estimates for the mean force error in both the direct and slider conditions were obtained. Reliability estimates (Spearman–Brown corrected) were Direct: r_SB_ = 0.94 and Slider: r_SB_ = 0.86.

**Table 1:** Participant demographics and self-reported psychological measures

	Mean (SD) or *N* / %
Age	24.51 (8.71)
Female gender	95 / 69%
Anxiety	5.41 (5.02)
Depression	6.32 (5.86)
Positive affect	29.25 (8.26)
Negative affect	19.97 (7.85)
Delusional ideation	5.14 (3.73)

Gender presented as count and percentage. Continuous variables presented as means and standard deviations. Anxiety = GAD-7 (0–21), Depression = PHQ-9 (0–27), Delusional ideation. = PDI-21 Scale-21 (0–21), and Positive/negative affect = PANAS (10–50)

### Determine the stability of the mean force error across different replications of the target force

Table 2 displays the mean force error and standard deviations by target force, condition, and number of force replications. The mean force error values are invariant across the 8, 6, 4, and 2 replications of the target force. Table 3 displays the formal contrast of the mean force error with differing target force replication (contrast column), as well as direct comparison of the reference replication (8 replications per force) with the reduced replications (2, 4 and 6 replications). No significant differences were observed, indicating that a reduction of the trial replications would not compromise our estimate of sensory attenuation in the force-matching task.

**Table 2:** Mean force error by condition, target force and number of replications

	8 Replications	6 Replications	4 Replications	2 Replications
Direct: 1 N	0.49 (0.62)	0.49 (0.60)	0.49 (0.61)	0.50 (0.70)
Direct: 1.5 N	0.52 (0.76)	0.53 (0.74)	0.52 (0.75)	0.51 (0.84)
Direct: 2 N	0.53 (0.87)	0.53 (0.85)	0.55 (0.86)	0.53 (0.92)
Direct: 2.5 N	0.39 (0.92)	0.41 (0.88)	0.40 (0.92)	0.36 (1.03)
Slider: 1 N	– 0.12 (0.27)	– 0.12 (0.24)	– 0.12 (0.28)	– 0.11 (0.31)
Slider: 1.5 N	– 0.20 (0.34)	– 0.19 (0.31)	– 0.20 (0.34)	– 0.21 (0.40)
Slider: 2 N	– 0.31 (0.41)	– 0.31 (0.39)	– 0.31 (0.40)	– 0.32 (0.48)
Slider: 2.5 N	– 0.49 (0.48)	– 0.49 (0.46)	– 0.50 (0.45)	– 0.48 (0.54)

Values presented as means (standard deviations). 8 replications are standard for the paradigm

**Table 3:** ANOVA of mean force error by condition, target force and number of replications

	Combined	2 vs. 8 replications	4 vs. 8 replications	6 vs. 8 replications
Direct: 1 N	F(3, 411) = 0.12, *p* = 0.95	F(3, 411) = 0.13, *p* = 0.72	F(3, 411) = 0.01, *p* = 0.90	F(3, 411) = 0.04, *p* = 0.84
Direct: 1.5 N	F(3, 411) = 0.24 *p* = 0.87	F(3, 411) = 0.51, *p* = 0.48	F(3, 411) = 0.03, *p* = 0.86	F(3, 411) = 0, *p* = 0.98
Direct: 2 N	F(3, 411) = 0.2, *p* = 0.90	F(3, 411) = 0, *p* = 0.96	F(3, 411) = 0.47, *p* = 0.49	F(3, 411) = 0.09, *p* = 0.77
Direct: 2.5 N	F(3, 411) = 1.09, *p* = 0.35	F(3, 411) = 1.95, *p* = 0.16	F(3, 411) = 0.04, *p* = 0.85	F(3, 411) = 0.08, *p* = 0.78
Slider: 1 N	F(3, 411) = 0.31, *p* = 0.82	F(3, 411) = 0.05, *p* = 0.83	F(3, 411) = 0.34, *p* = 0.56	F(3, 411) = 0.27, *p* = 0.61
Slider: 1.5 N	F(3, 411) = 1.07, *p* = 0.36	F(3, 411) = 1.44, *p* = 0.23	F(3, 411) = 0.1, *p* = 0.75	F(3, 411) = 0.3, *p* = 0.58
Slider: 2 N	F(3, 411) = 0.19, *p* = 0.90	F(3, 411) = 0.54, *p* = 0.46	F(3, 411) = 0.09, *p* = 0.76	F(3, 411) = 0.2, *p* = 0.65
Slider: 2.5 N	F(3, 411) = 0.4, *p* = 0.75	F(3, 411) = 0.02, *p* = 0.90	F(3, 411) = 0.77, *p* = 0.38	F(3, 411) = 0.07, *p* = 0.79

Within-subject repeated measure ANOVA, using the mean error as the dependant variable with number of replications as the independent variable. A total of 8 analyses were conducted which included an omnibus test of simultaneous equality of all number of replications of the target force, as well as specific contrasts of 8 replications with each of the 2, 4, and 6 replications. These contrasts were conducted for each condition and target force

Split-half reliability estimates (Spearman–Brown corrected) for the mean force error in both the direct and slider conditions and each number of replications were obtained. While all reliability estimates across differing replications are acceptable, there is a general increase in reliability with higher replications of the target force: two replications; Direct: r_SB_ = 0.88 and Slider: r_SB_ = 0.74, four replications; Direct: r_SB_ = 0.91 and Slider: r_SB_ = 0.81 and six replications; Direct: r_SB_ = 0.92 and Slider: r_SB_ = 0.84.

### Aim 2 – Determine the optimal time window for the extraction of the mean force error estimates

**Fig. 2:**
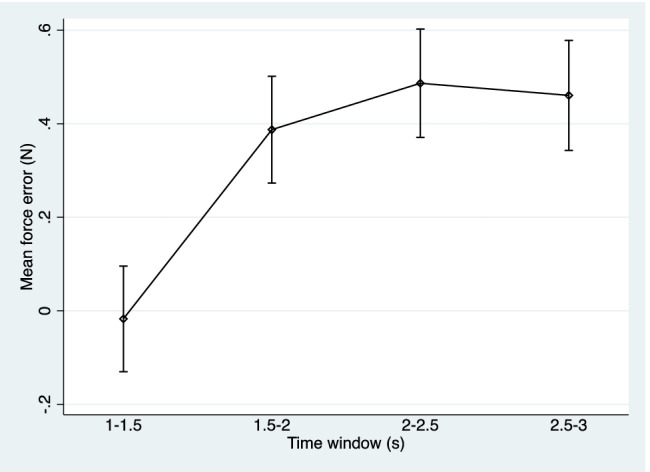
Confidence interval plot of mean force error and 95% confidence intervals across different time windows in the direct condition

**Fig. 3:**
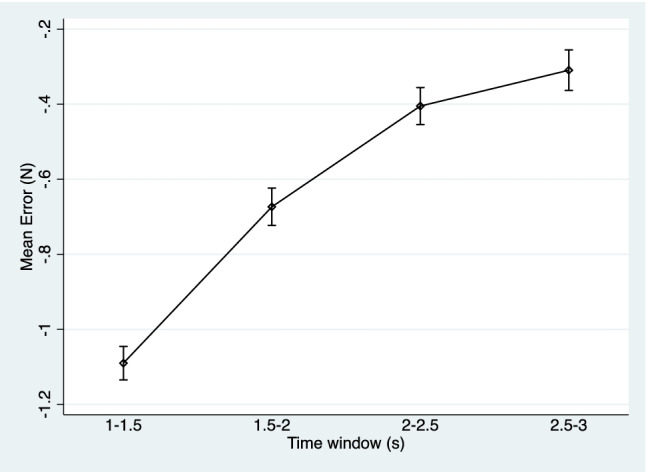
Confidence interval plot of mean force error and 95% confidence intervals across different time windows in the slider condition

Table 4 displays the mean force error and standard deviation across the four time windows extracted from the period of time the participants matched the target force after the go signals (1–1.5 s, 1.5–2 s, 2–2.5 s, and 2.5–3 s). In the direct condition, the mean force error increased, and participants became less accurate as the time window increased. In the slider condition, the mean force error increased, and participants become more accurate as the time window increased. Figures 2 and 3 illustrate this trend via confidence interval plots. Statistically significant differences between the time windows were observed in both conditions (Table 5). In the direct condition, comparisons with the reference time window (2.5–3 s) identified consistent and statistically significant differences with the 1–1.5-s and 1.5–2-s intervals (expect for one model regarding the 1N target force), while no statistically significant differences were noted between the models comparing 2.0–2.5- and 2.5–3-s time windows. This indicates in the direct condition, either the 2–2.5- or 2.5–3-s time windows would be suitable for the extraction of the mean force error. In the slider condition, the comparisons with the reference time window identified statistically significant differences in all models.

**Table 4:** Mean force error by condition, target force, and time window

	1.0–1.5 s	1.5–2.0 s	2.0–2.5 s	2.5–3.0 s
Direct: 1 N	0.23 (0.53)	0.45 (0.58)	0.50 (0.60)	0.48 (0.59)
Direct: 1.5 N	0.50 (0.64)	0.43 (0.67)	0.53 (0.69)	0.50 (0.70)
Direct: 2 N	– 0.08 (0.79)	0.40 (0.78)	0.53 (0.81)	0.49 (0.83)
Direct: 2.5 N	– 0.26 (0.87)	0.27 (0.86)	0.39 (0.85)	0.38 (0.88)
Slider: 1 N	– 0.55 (0.20)	– 0.32 (0.21)	– 0.18 (0.23)	– 0.14 (0.24)
Slider: 1.5 N	– 0.91 (0.25)	– 0.54 (0.28)	– 0.31 (0.29)	– 0.22 (0.32)
Slider: 2 N	– 1.24 (0.33)	– 0.76 (0.37)	– 0.45 (0.37)	– 0.34 (0.40)
Slider: 2.5 N	– 1.67 (0.36)	– 1.08 (0.43)	– 0.68 (0.44)	– 0.54 (0.49)

Values presented as means (standard deviations). 2.5–3.0 s is consistent for the paradigm

**Table 5:** ANOVA of mean force error by condition, target force and time window

	Combined	1.0–1.5 vs. 2.5–3.0	1.5–2.0 vs. 2.5–3.0	2.0–2.5 vs. 2.5–3.0
Direct: 1 N	F(3, 411) = 78.99, *p* < 0.001	F(3, 411) = 153.51, *p* < 0.001	F(3, 411) = 1.32, *p* = 0.25	F(3, 411) = 1.52, *p* = 0.21
Direct: 1.5 N	F(3, 411) = 144.77 *p* < 0.001	F(3, 411) = 300.56, *p* < 0.001	F(3, 411) = 7.62, *p* = 0.01	F(3, 411) = 0.86, *p* = 0.36
Direct: 2 N	F(3, 411) = 130.61, *p* < 0.001	F(3, 411) = 266.35, *p* < 0.001	F(3, 411) = 5.95, *p* = 0.02	F(3, 411) = 1.29, *p* = 0.26
Direct: 2.5 N	F(3, 411) = 156.35, *p* <.001	F(3, 411) = 335.00, *p* < 0.001	F(3, 411) = 10.37, *p* =.001	F(3, 411) = 0.20, *p* = 0.65
Slider: 1 N	F(3, 411) = 393.40, *p* < 0.001	F(3, 411) = 963.86, *p* < 0.001	F(3, 411) = 183.65, *p* < 0.001	F(3, 411) = 9.01, *p* = 0.003
Slider: 1.5 N	F(3, 411) = 618.91, *p* < 0.001	F(3, 411) = 1541.54, *p* < 0.001	F(3, 411) = 331.76, *p* < 0.001	F(3, 411) = 22.32, *p* < 0.001
Slider: 2 N	F(3, 411) = 774.97, *p* < 0.001	F(3, 411) = 1936.89, *p* < 0.001	F(3, 411) = 419.91, *p* < 0.001	F(3, 411) = 30.04, *p* < 0.001
Slider: 2.5 N	F(3, 411) = 858.21, *p* < 0.001	F(3, 411) = 2152.46, *p* < 0.001	F(3, 411) = 493.67, *p* < 0.001	F(3, 411) = 37.45, *p* < 0.001

Within-subject repeated measure ANOVA, using the mean error as the dependant variable with timeframe windows as the independent variable. A total of 8 analyses were conducted which included an omnibus test of simultaneous equality of all time windows, as well as specific contrasts of the 2.5–3.0s time window with each 1–1.5s, 1.5–2s and 2–2.5s time windows. These contrasts were conducted for each condition and target force

### Aim 3 - Evaluate learning or fatigue effects associated with matching a force over the course of the experiment

**Fig. 4:**
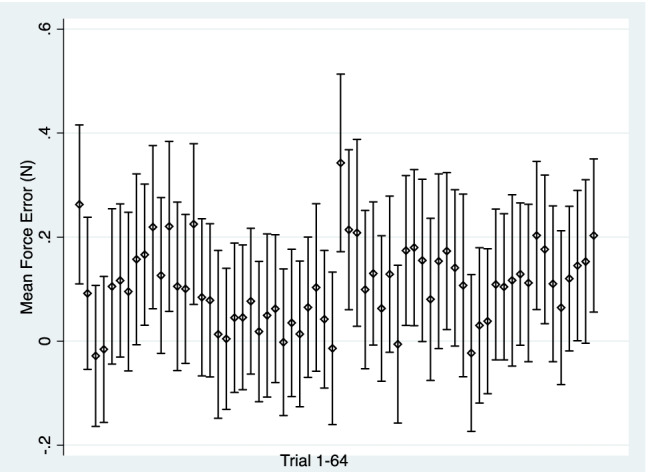
Confidence interval plot of mean force error and 95% confidence intervals across trial sessions (1–64). Conditions were counterbalanced across participants. No observable trend or learning effects are identified

**Fig. 5:**
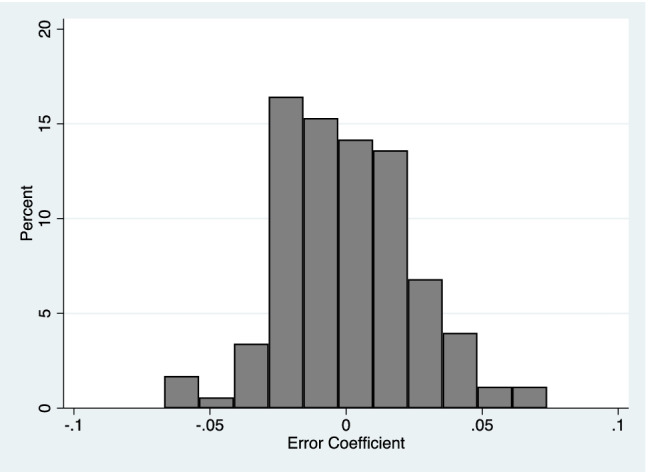
Histogram of the error coefficient showing the individual variation between participants. The error coefficient represents the slope of the mean force error of 64 trails, calculated via a linear regression analysis for each subject

There was minimal change in the mean force error across all 64 trials. The mean force error coefficient was 0.0004 (SD = 0.02) and the average statistical significance level for the coefficient was *p* = 0.08 (SD = 0.19). Figure 4 displays a confidence interval plot of the mean force error of all 64 trials and highlighted no trend: participants, on average, did not improve or worsen in performance. Further, Fig. 5 displays a histogram of the mean force error coefficient, which was largely centred around 0. Specifically, 95% of the coefficients lie between – 0.05 and 0.05. As this represents approximately 10% of the overall average mean force error, the number of individuals with error coefficients greater than 0.05 or less than –0.05 would be considered of minimal practical relevance to the task.

### Aim 4 – Determine whether learning or fatigue effects are moderated by any psychological traits of the participant

Spearman’s correlations were conducted to determine whether trends during the experimental process were associated with psychological traits. All correlations were negligible and non-statistically significant indicating no evidence of a relationship between the mean force error coefficient and psychological measures: Depression (*rho* = 0.08, *p* = 0.32), anxiety (*rho* = 0.03, *p* = 0.69), negative effect (*rho* = 0.002, *p* = 0.98), positive effect (*rho* = 0.003, *p* = 0.97) and delusional ideation (*rho* = – 0.03, *p* = 0.73).

## Discussion

In this paper, we sought to evaluate a number of key points of methodology in the experimental protocol of force-matching paradigm. These details are crucial to facilitating reliable and replicable estimates of an individual’s level of sensory attenuation. We identified three main findings from this work. Firstly, the number of replications of the target forces may be reduced from eight to two without compromising the estimate of sensory attenuation. Secondly, the optimal time window for the extraction of the matched force measurements is 2.5–3 s. Finally, there was no evidence of learning or fatigue effects over the course of the experimental protocol.

Consistent to previous research using our labs force-matching device (McNaughton et al., 2021; McNaughton et al., 2022) and others (Palmer et al., 2016; Shergill et al., 2014; Teufel et al., 2010; Walsh et al., 2011; Wolpe et al., 2016), we identified an overestimation of the matched force in the direct condition. This indicates the fundamental sensorimotor attenuation phenomenon, whereby attenuation of sensations arising from one’s own action depends on the integration of predictive signals and sensory feedback (Bays et al., 2006). This is in comparison to a more accurate estimation of the target force in the slider condition, which is hypothesised to be related to reduced efference copy signals used in the sensorimotor perception of external forces.

Determining the optimal number of target force replications necessary for a reliable estimate of sensory attenuation has a direct influence on the future of this task. Previous research has consistently proposed eight replications for any measure of sensory attenuation (Parees et al., 2014; Shergill et al., 2014; Teufel et al., 2010; Wolpe et al., 2016) leading to a total of 64 trials per participant in the experimental protocol. This results in a task which can take up to 45 mins and is cognitively demanding, from both the perspective of the investigator manually controlling the device, as well as by participants to feel the target force, remember that feeling, and reproduce the force in a timely manner. Our results suggest that reducing the number of replications of the target force to 4 or 6 may reduce the overall time of the paradigm and cognitive load of the participants and the investigators, without compromising the sensory attenuation estimate.

Regarding the optimal time window to extract the matched force, previous research has reported both the 2–2.5-s (Shergill et al., 2014; Wolpe et al., 2016) and 2.5–3-s windows (McNaughton et al., 2021; Palmer et al., 2016; Parees et al., 2014). Regarding our findings, in the direct condition, as the time window increased, participants’ mean force error increased until the 2–2.5-s time window and then plateaued, suggesting extraction from 2 s after the go signal would be suitable. This is reflective of the time to react to the go signal and begin to apply pressure through the transducer, before intrinsically maintaining a force which was perceived to be similar to the target force. These findings are consistent with those described in Voss et al. (2007), showing a similar matched force profile over time. In the slider condition, as the time window increased, the participants’ mean force error continued to become more accurate, suggesting the 2.5–3-s time window as the most suitable. Thus, in order to maintain consistency of the time-interval across both conditions, our estimate of sensory attenuation is optimally taken from the 2.5–3-s time window. Why this trend difference between conditions occurs may be due to subtle lag time between moving the slider and the corresponding downward force sensed by participants. This may be related to an added linearising function of the potentiometer output, which converts the potentiometer output to the corresponding change of position of the lever. The resulting coefficient of the fourth-degree polynomial are the basis of this linearising function. This is in comparison to more immediate and forceful sensory feedback when participants use their opposite finger in the direct condition.

Finally, it was of interest to determine if any learning effects or fatigue occurred over the course of the experiment, thus compromising the estimate of sensory attenuation towards the end of the task. We identified limited evidence of worsening or improving performance over the course of the 64 trials, suggesting a stable estimate of sensory attenuation. Specifically, we identified very few individuals (< 5%) who had a slope greater than 0.05 or less than –0.05, which is less than 10% of the average mean force error. We determined the magnitude and frequency of these findings to be practically irrelevant to the overall task. Finally, psychological measures (depression, anxiety, positive and negative effect, and delusional ideation) were unrelated to any trend.

A limitation of this work is that the sensory attenuation estimates derived from the mean force error are only applicable to our device. We have previously shown differences in force error estimates between devices (McNaughton et al., 2021) and this should be taken into account when planning future experiments utilising this task.

The current study systematically evaluated the methodology underling the force-matching task, providing positive results that make the running of this experimental paradigm more efficient and speak to the reliability of the task. The number of replications of the target force may be reduced without compromising the estimate of sensory attenuation, the optimal time window to extract the matched force is 2.5–3 s after the go signal, and performance throughout the task is stable. These results should be considered when developing future research that incorporates the force-matching task.

## Data Availability

Data and analysis code is available from the Macquarie University data repository. 10.25949/19694998.v1.

## Code availability

Device related code is available and reported elsewhere.
